# Language Individuation and Marker Words: Shakespeare and His Maxwell's Demon

**DOI:** 10.1371/journal.pone.0066813

**Published:** 2013-06-27

**Authors:** John Marsden, David Budden, Hugh Craig, Pablo Moscato

**Affiliations:** 1 Centre for Bioinformatics, Biomarker Discovery & Information-Based Medicine, The University of Newcastle, Callaghan, New South Wales, Australia; 2 Centre for Literary and Linguistic Computing, The University of Newcastle, Callaghan, New South Wales, Australia; Max Planck Institute for the Physics of Complex Systems, Germany

## Abstract

**Background:**

Within the structural and grammatical bounds of a common language, all authors develop their own distinctive writing styles. Whether the relative occurrence of common words can be measured to produce accurate models of authorship is of particular interest. This work introduces a new score that helps to highlight such variations in word occurrence, and is applied to produce models of authorship of a large group of plays from the Shakespearean era.

**Methodology:**

A text corpus containing 55,055 unique words was generated from 168 plays from the Shakespearean era (16th and 17th centuries) of undisputed authorship. A new score, CM1, is introduced to measure variation patterns based on the frequency of occurrence of each word for the authors John Fletcher, Ben Jonson, Thomas Middleton and William Shakespeare, compared to the rest of the authors in the study (which provides a reference of relative word usage at that time). A total of 50 WEKA methods were applied for Fletcher, Jonson and Middleton, to identify those which were able to produce models yielding over 90% classification accuracy. This ensemble of WEKA methods was then applied to model Shakespearean authorship across all 168 plays, yielding a Matthews' correlation coefficient (MCC) performance of over 90%. Furthermore, the best model yielded an MCC of 99%.

**Conclusions:**

Our results suggest that different authors, while adhering to the structural and grammatical bounds of a common language, develop measurably distinct styles by the tendency to over-utilise or avoid particular common words and phrasings. Considering language and the potential of words as an abstract chaotic system with a high entropy, similarities can be drawn to the Maxwell's Demon thought experiment; authors subconsciously favour or filter certain words, modifying the probability profile in ways that could reflect their individuality and style.

## Introduction

Although authors are required to adhere to the grammatical and structural rules dictated by a written language, each author is able to develop a highly individual style within this framework [1], [2]. One form this language individuation takes is systematic variation in the relative frequencies of particular words and phrases. This variation in turn provides a basis for the accurate classification of authorship. The idea that this sort of variation occurs even in the use of the most common words, and that frequencies of these words could serve for authorship attribution, goes back to the 1960s (specifically, the statistical work of Ellegard on a set of anonymous eighteenth-century published letters [3] and of Mosteller and Wallace on the jointly authored Federalist papers [4]), but was developed to a regular technique by Burrows in the 1980s. Burrows pioneered the use of multivariate techniques like Principal Components Analysis on sets of frequencies of very common words to attribute disputed texts [5], [6], and similar methodologies have since been widely used [7]–[9].

Researchers have also explored the usefulness for attribution of slightly less common words, which tend to be lexical words rather than function words, and of very rare words [10]–[12]. In general, authorship study using quantitative methods (most often relying on word frequencies, but also exploiting letter and word 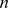-grams, and the frequency of punctuation) is now well established and has been the subject of several reviews [13]–[15]. This field is variously referred to as *stylometry* and *computational stylistics*.

The tendency recently has been to use longer and longer lists of marker words [16], as well as word sequences and collocations[14], [17], [18], and it may be useful to focus on the degree of distinctiveness in the frequencies of a subset of the very common words between authors, and their resulting power to provide efficient classification by author. It is also worth noting that while in many operations with natural language (such as topic detection and text searching), the usual practice is to discard the most common words (so-called ‘stopwords’ [19], [20]). In contrast, these stopwords are the focus of the present analysis, and the constant added to the CM1 function, described below, provides a bias toward these very common words. They prove to be highly discriminating for the authors tested.

**Figure 1:**
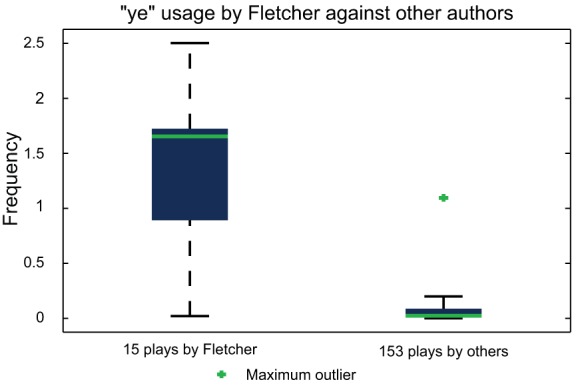
Observed frequency of Fletcher's usage of the word ‘
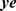
’ in his 15 plays, compared to that of the 153 plays by other authors in the text corpus dataset. A significantly higher frequency of ‘
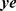
’ usage by Fletcher is demonstrated, indicating ‘
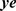
’ as an appropriate choice of marker to assist in the classification of his plays. Fletcher's predilection for the word ‘
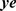
’ has been previously shown by Hoy [21].

The use of the word ‘
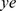
’ by seventeenth-century playwrite John Fletcher provides a striking example of idiosyncratic word usage, as previously demonstrated by Hoy [21]. This contribution focuses on the identification of such marker words and introduces a new score, CM_–_1, that allows for the identification of patterns of variation based on the relative frequency of word usage present in a text corpus dataset of 168 plays from the Shakespearean era. As an example, the CM_–_1 score confirms Fletcher's tendency to use ‘
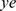
’; Figure 1 demonstrates the observed frequencies for ‘
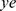
’ in his 15 plays, plotted against 153 plays by other authors. Despite the wide range of frequencies, the spectacularly high median demonstrates ‘
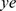
’ to be an ideal marker for modeling Fletcher's authorship if restricted to a single word. More stylistic characteristics could be revealed by extending from one word to many, providing more robust authorship characterisation.

**Figure 2:**
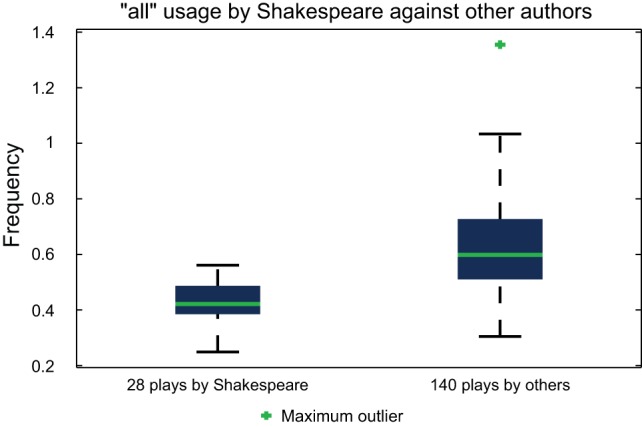
Observed frequency of Shakespeare's usage of the word ‘
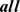
’ in his 28 plays, compared to that of the 140 plays by other authors in the text corpus dataset. A significantly lower frequency of ‘
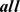
’ usage by Shakespeare is demonstrated, indicating ‘
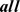
’ as an appropriate choice of marker to assist in the classification of his plays.

For some authors, such as Shakespeare, negative markers seem to yield more accurate language individuation. An interesting consequence of this observation is the notion that some writers are better defined by words they under-utilise, rather than those which they prefer. This has been discussed but not widely supported by in-depth analysis [12], [22], and is one of the motivations of the present work. This variation in frequency may result from the conscious or subconscious censoring of particular words when authors choose formulations for their writing, or rather may be an implicit indicator of a preference for constructions or stances which reduce the need for these words. Figure 2 demonstrates the observed frequencies for Shakespeare's usage of ‘
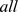
’ in his 28 considered plays, plotted against 140 plays by other authors.

In this work, the CM_–_1 score (introduced in the Materials and Methods section) was applied to identify the 20 highest and 20 lowest scoring marker words for John Fletcher, Ben Jonson and Thomas Middleton. 50 methods from the popular open source data mining and machine learning package WEKA [23] were utilised to produce models of authorship based on these markers, with performance evaluated in terms of Matthews' correlation coefficient. An ensemble of the best performing WEKA methods were finally applied to the classification of Shakespearean plays, considering only the 20 highest and 20 lowest CM1 scoring marker words generated from these 28 works (and the 140 plays by other authors).

## Materials and Methods

### Text Corpus Dataset

A text corpus containing 168 plays from the Shakespearean era was utilised for this work, containing texts of unambiguous authorship from the 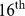 and 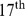 centuries. From this corpus, the Intelligent Archive by Craig and Whipp [24] was applied to generate a set of approximately 55,055 unique words, composed of every word from across all 168 plays. The Intelligent Archive is a software tool which can be utilised to create sub-corpora and generate counts of word-forms according to a parameterised user input, taking into account the variations in spelling commonly found in 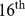 and 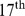 century plays, in addition to facilitating disambiguation of words by both context and frequency. For each play, the frequency of each of the aforementioned 55,055 words was calculated and stored in the form of a 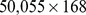 matrix; a total of in excess of nine million word usage statistics.

### Methods

Given the dataset generated by the Intelligent Archive, a method of filtering the full set 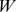 of 55,055 unique words to determine a set of marker words (those which distinguish one author's work from that of the others) is required. Four authors (John Fletcher, Ben Jonson, Thomas Middleton and William Shakespeare) were chosen, as they account for the largest number of plays in the corpus dataset. One commonly accepted method of filtering such a dataset is Welch's 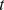-test,
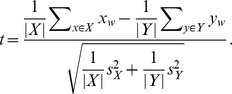



This adaptation of Student's 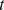-test allows for samples of unequal variance, but otherwise treats the two sample partitions 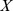 and 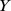 of observations as homogeneous overall, with the moderator chosen as the standard deviation of the combined set [25]. This assumption does not apply in the case of identifying marker words; one set contains the works of a single author, whereas the other contains the combined works of a large number of authors. Instead, a new method (the CM1 score) was devised, with a moderator that considers the range of values rather than the combined standard deviation.

Let 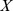 and 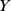 be a partition of the set 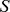 of all plays in the dataset, meaning that 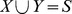 and 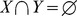. Let 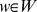 be one of the words used and let 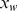 be the frequency of occurrence of the word 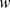 in play 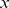 of a given target author. Let 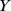 be the set of all other plays not written by this author and, analogously, let 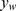 be the frequency of occurrence of the word 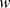 in play 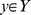. The CM1 score is then defined as
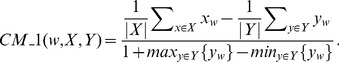
(1)


The CM_–_1 scores of all words were investigated in four partitions of the text corpus dataset, in which the target author plays 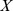 were known to be the work of Fletcher, Jonson, Middleton or Shakespeare. From the original set of approximately 55,055 words, the 20 highest and 20 lowest CM_–_1-scoring marker words were selected and sorted according to their CM_–_1 scores.

Like the 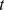-test, the CM_–_1 score calculates the difference between means; however, the score is moderated by the range of values for the non-authorial reference set 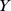, rather than the combined standard deviation of 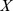 and 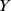. The constant unity is added to the moderator so that variables of higher frequency will tend to result in higher scores, arising from the a priori view that more frequent variables will result in more reliable markers.

Having calculated the 20 lowest and 20 highest marker words for Fletcher, Jonson and Middleton, 50 machine learning methods from the open source data mining and machine learning package WEKA [23] were utilised to produce mathematical models of authorship. Table 1 lists all of the methods considered, along with their respective types, as categorised in WEKA version 3.6.4. In each case, a 10-by-10 fold cross validation of each author's marker words was performed, with the Matthews' correlation coefficient of the classification calculated. In data mining and machine learning, a 10-by-10 fold cross validation involves randomly partitioning the original dataset into 10 equal sized subsets; 9 used for training data, and the remaining subset reserved for evaluation. Evaluation is repeated 10 times, such that each subset is utilised exactly once for this purpose [26].

**Table 1:** List of methods and their types.

Method	Type	Method	Type
BayesianLogisticRegression	bayes	MultiBoostAB	meta
BayesNet	bayes	RandomCommittee	meta
ComplementNaiveBayes	bayes	RandomSubSpace	meta
NaiveBayes	bayes	RotationForest	meta
NaiveBayesUpdateable	bayes	Stacking	meta
Logistic	functions	ConjunctiveRule	rules
MultilayerPerceptron	functions	DecisionTable	rules
RBFNetwork	functions	DTNB	rules
SimpleLogistic	functions	JRip	rules
SMO	functions	NNge	rules
Spegasos	functions	OneR	rules
VotedPerceptron	functions	PART	rules
IB1	lazy	Ridor	rules
IBk	lazy	ZeroR	rules
KStar	lazy	ADTree	trees
LWL	lazy	BFTree	trees
AdaBoostM1	meta	FT	trees
AttributeSelectedClassifier	meta	J48	trees
Bagging	meta	LADTree	trees
ClassificationViaClustering	meta	LMT	trees
ClassificationViaRegression	meta	NBTree	trees
Dagging	meta	RandomForest	trees
Decorate	meta	RandomTree	trees
END	meta	REPTree	trees
LogitBoost	meta	SimpleCart	trees

List of methods utilised in this work and their respective types, as categorised in WEKA [23] version 3.6.4. No manual tuning of parameters was undertaken, with all parameters set to their default values.

A subset of the original 50 WEKA methods (those generating models that yielded a Matthews' correlation coefficient of over 90% for Fletcher, Jonson and Middleton) were selected for application to Shakespeare. This ensemble of WEKA methods was used to generate mathematical models of Shakespeare's writing style, considering only the 20 highest and 20 lowest CM_–_1 scoring marker words. The sensitivity (probability of determining the text to be written by the considered author, given that it was) and specificity (probability of determining the text to have not been written by the considered author, given that it was written by another) were calculated for a 10-by-10 fold cross validation of these models, and finally combined into the Matthews' correlation coefficient [27], [28] (which is the preferred approach for preserving classification performance for binary classification in an unbiased way).

The Matthews' correlation coefficient, 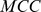, is defined as
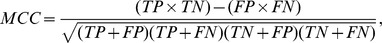
where 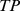 and 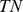 are the number of *true positives* and *true negatives* respectively (correct classification of an author having written (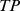) or not written (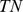) a given play), and 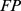 and 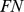 are the number of *false positives* and *false negatives* respectively (incorrectly determining a play as having been written by some author (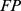), or failing to recognise that it has (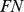)).

## Results

### Selection of Marker Words using CM_–_1 Score

The CM_–_1 score was calculated for all 55,055 unique words present in the text corpus dataset, for Fletcher, Jonson, Middleton and Shakespeare. The 50 highest and 50 lowest scoring words were ranked for each author, with the 20 highest and 20 lowest presented in Tables 2 and 3.

**Table 2:** 20 highest ranking words by CM1 score (presented in descending order of score), for Fletcher, Jonson, Middleton and Shakespeare.

Fletcher	Jonson	Middleton	Shakespeare
have	any	so[adverbDegree]	will[noun]
her[personalPronoun]	in[adverb]	master	thee
will[verb]	good	me	you
there	have	am	did
dare	aye	gentleman	that[conjunctive]
sure	of	have	do
now	they	one	good
a	yes	widow	not
your	them	upon[preposition]	speak
she	your	for[preposition]	come
it	here	o	hath
no[adjective]	and	there	go
am	a	never	say
do	it	I	me
are	him	you	him
and	do	now	the
me	the	a	so[adverbManner]
I	he	is	he
too	you	it	is
ye	or	that[demonstrative]	thou

**Table 3:** 20 lowest ranking words by CM_–_1 score (presented in descending order of score), for Fletcher, Jonson, Middleton and Shakespeare.

Fletcher	Jonson	Middleton	Shakespeare
in[preposition]	my	and	all
of	thou	hath	to[infinitive]
the	me	that[conjunction]	now
my	thy	with	ye
that[conjunction]	to[preposition]	the	can
hath	for[conjunction]	this	may
to[infinitive]	from	thou	are
by[preposition]	lord	on[preposition]	for[preposition]
his	thee	do	must
lord	king	as	see
with	death	thy	their
to[preposition]	that[relative]	king	your
which[relative]	ye	or	only
as	shall	ye	yet
thy	that[conjunction]	these	a
but	our[truePlural]	doth	they
than	heaven	of	or
you	yet	their	yes
doth	never	god	still
aye	blood	thus	but

**Figure 3:**
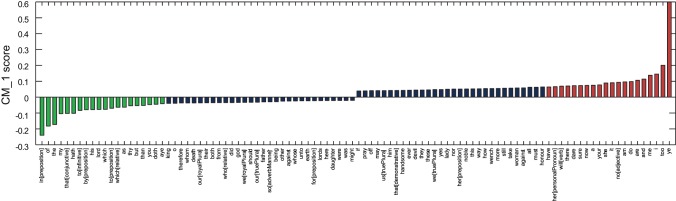
CM_–_1 scores for the 50 highest and 50 lowest ranked words for Fletcher, based on the 168 plays in the text corpus dataset. The 20 highest and 20 lowest ranked words are shown in red and green respectively, and are presented in Tables 2 and 3. As expected, the CM_–_1 score for ‘
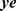
’ is significantly higher than that of any other marker word.

Figure 3 demonstrates the CM_–_1 score for the 50 highest and 50 lowest scoring marker words for John Fletcher, with the 20 highest and 20 lowest ranked words shown in red and green respectively. Fletcher's plays account for 15 of the 168 present in the text corpus dataset. ‘
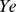
’ is shown to dominate as a positive marker, with the lowest scoring negative markers included ‘
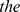
’, ‘
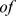
’ and the prepositional form of ‘
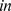
’.

**Figure 4:**
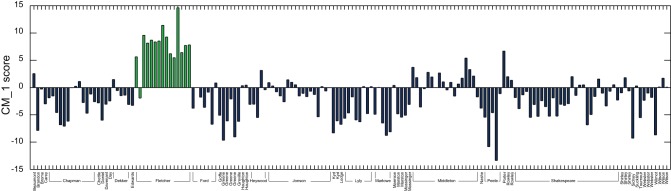
Difference between the cumulative CM_–_1 scores for Fletcher's 20 highest and 20 lowest scoring marker words, as presented in Tables 2 and 3. Fletcher's plays are highlighted in green. It is observed that the majority of his plays score considerably higher than the majority of plays by the other authors. One notable exception, *The Faithful Shepherdess*, is considered to be of a significantly different genre to the remainder of Fletcher's plays, and has been omitted in two previous studies attempting to identify his stylistic signature [21], [36].

The difference between the probabilities of Fletcher's 20 highest and 20 lowest scoring marker words was calculated across all 168 plays from the text corpus dataset, with the results presented in Figure 4. All of Fletcher's plays score strong positive results against these markers, with the exception of *The Faithful Shepherdess*.

**Figure 5:**
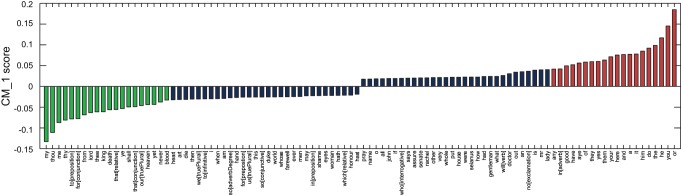
CM_–_1 scores for the 50 highest and 50 lowest ranked words for Jonson, based on the 168 plays in the text corpus dataset. The 20 highest and 20 lowest ranked words are shown in red and green respectively, and are presented in Tables 2 and 3. CM_–_1 ranks ‘
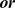
’ and ‘
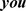
’ as words that Jonson distinctively overuses, in contrast to ‘
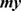
’ and ‘
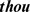
’, which are distinctively underused.

**Figure 6:**
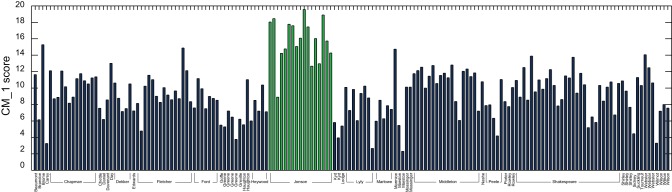
Difference between the cumulative CM_–_1 scores for Jonson's 20 highest and 20 lowest scoring marker words, as presented in Tables 2 and 3. Jonson's plays are highlighted in green. Although not as evident as with Fletcher, Jonson's plays demonstrate an overall higher score than the majority of plays by the other authors. The worst scoring of Jonson's plays, *The Case is Altered*, is generally regarded as a stylistic anomaly among his works [35].

Figure 5 demonstrates the CM1 score for the 50 highest and 50 lowest scoring marker words for Ben Jonson, whose plays account for 17 of the 168 considered. Jonson's positive markers include ‘
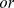
’ and ‘
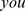
’, with ‘
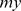
’ and ‘
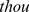
’ dominating the negative markers. The difference between Jonson's 20 highest and 20 lowest scoring marker words is presented in Figure 6.

**Figure 7:**
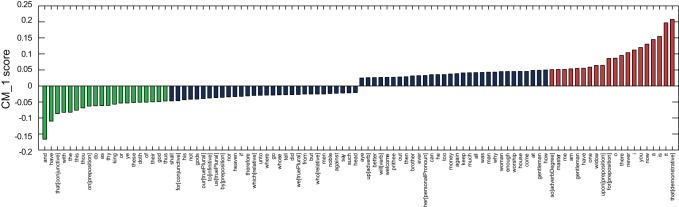
CM_–_1 scores for the 50 highest and 50 lowest ranked words for Middleton, based on the 168 plays in the text corpus dataset. The 20 highest and 20 lowest ranked words are shown in red and green respectively, and are presented in Tables 2 and 3. CM_–_1 ranks ‘
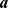
’, ‘
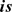
’, ‘
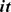
’ and the demonstrative form of ‘
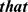
’ among the words that Middleton distinctively overuses; ‘
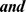
’ is ranked amongst the words that Middleton underuses, as opposed to plays by Jonson, for which ‘
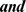
’ is a strong positive marker.

**Figure 8:**
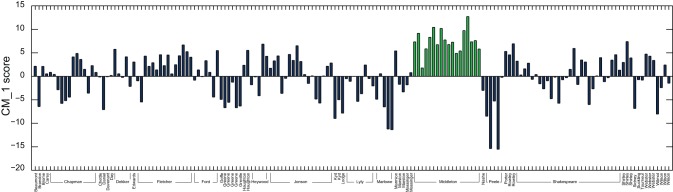
Difference between the cumulative CM_–_1 scores for Middleton's 20 highest and 20 lowest scoring marker words, as presented in Tables 2 and 3. Eight of these marker words appeared among the ten word-variables determined earlier by Craig [34] (by discriminant analysis). Middleton's plays are highlighted in green. It is observed that the majority of his plays score higher than the majority of plays by the other authors. The worst scoring of Middleton's plays, *A Game at Chess*, is unusual stylistically among his works, being a satire on contemporary international politics [32], [33].

Figure 7 demonstrates the CM_–_1 score for the 50 highest and 50 lowest scoring marker words for Thomas Middleton, whose plays account for 18 of the 168 considered. Middleton's positive markers include ‘
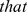
’, ‘
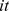
’, ‘
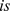
’ and ‘
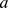
’, with his negative markers including ‘
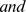
’ and ‘
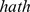
’. The difference between Middleton's 20 highest and 20 lowest scoring marker words is presented in Figure 8.

**Figure 9:**
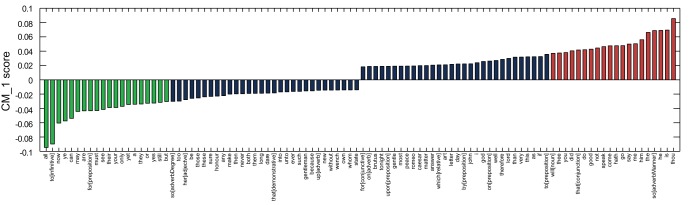
CM_–_1 scores for the 50 highest and 50 lowest ranked words for Shakespeare, based on the 168 plays in the text corpus dataset. The 20 highest and 20 lowest ranked words are shown in red and green respectively, and are presented in Tables 2 and 3. CM_–_1 ranks ‘
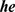
’, ‘
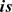
’ and ‘
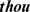
’ as words that Shakespeare distinctively overuses, in contrast to ‘
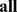
’ (as discussed by Craig [29]), ‘
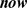
’ and the infinitive form of ‘
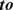
’, which are distinctively underused.

The final author considered was William Shakespeare, with Figure 9 demonstrating the CM1 score for his 50 highest and 50 lowest scoring words. Shakespeare's plays are the most well represented in the dataset, accounting for 28 of the total 168 contained in the text corpus dataset. Shakespeare's highest scoring marker word is ‘
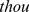
’, with his lowest ranking words including ‘
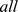
’ (matching a previous discussion by Craig [29]) and the infinitive version of ‘
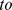
’.

**Figure 10:**
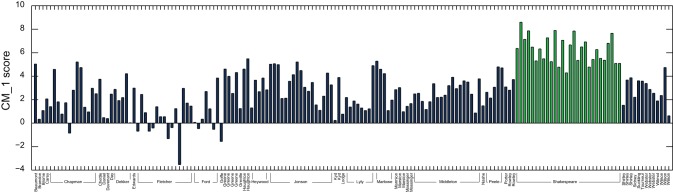
Difference between the cumulative CM_–_1 scores for Shakespeare's 20 highest and 20 lowest scoring marker words, as presented in Tables 2 and 3. Shakespeare's plays are highlighted in green. It is observed that the majority of his plays score higher than by other authors, although the overall range of values is lower than for Fletcher, Jonson and Middleton. This supports previous research suggesting that Shakespeare generally adheres to the norms of the work of his peer group [30].

Finally, the difference between Shakespeare's 20 highest and 20 lowest scoring marker words was calculated across all 168 plays from the text corpus dataset, with the results presented in Figure 10. Overall, Shakespeare's plays are demonstrated to rank considerably higher than those by other authors.

### Selection of Modelling Methods from CM_–_1 Features

**Figure 11:**
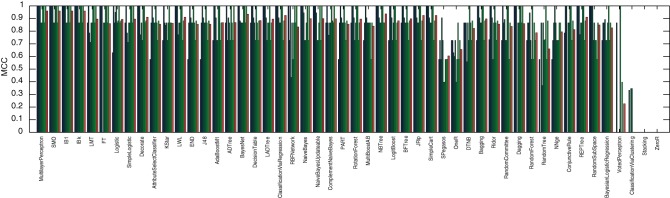
Authorship classification performance of 50 methods evaluated in terms of Matthews' correlation coefficient for Fletcher, resulting from a 10-by-10 fold cross validation of his 20 highest and 20 lowest CM_–_1 scoring marker words. The results of individual folds are presented in blue/green, with the average performance for each method in red.

**Figure 12:**
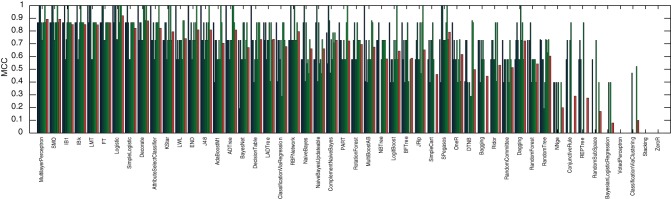
Authorship classification performance of 50 methods evaluated in terms of Matthews' correlation coefficient for Jonson, resulting from a 10-by-10 fold cross validation of his 20 highest and 20 lowest CM_–_1 scoring marker words. The results of individual folds are presented in blue/green, with the average performance for each method in red.

**Figure 13:**
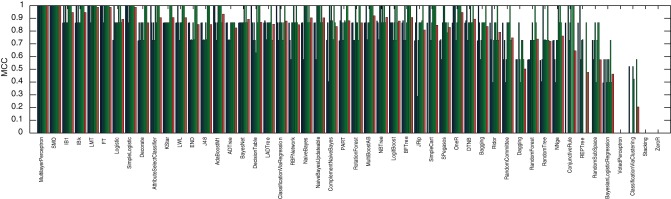
Authorship classification performance of 50 methods evaluated in terms of Matthews' correlation coefficient for Middleton, resulting from a 10-by-10 fold cross validation of his 20 highest and 20 lowest CM_–_1 scoring marker words. The results of individual folds are presented in blue/green, with the average performance for each method in red.

Considering only the 20 highest and 20 lowest CM_–_1 marker words for Fletcher, Jonson and Middleton, and applying 50 methods from the popular open source data mining and machine learning package WEKA [23], a 10-by-10 fold cross-validation was performed. For each method, the performance of each fold was evaluated in terms of the Matthews' correlation coefficient to identify those that perform well with the pre-selection of markers based on the CM_–_1 score. These results, along with the mean performance for each method, are presented in Figures 11, 12 and 13, for Fletcher, Jonson and Middleton respectively.

**Figure 14:**
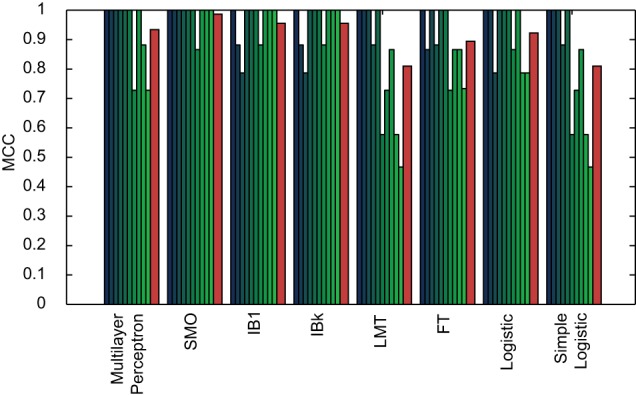
Authorship classification performance of 8 methods evaluated in terms of Matthews' correlation coefficient for Shakespeare, resulting from a 10-by-10 fold cross validation of his 20 highest and 20 lowest CM_–_1 scoring marker words. These 8 methods were selected as those which yielded the best classification performance for Fletcher, Jonson and Middleton. The results of individual folds are presented in blue/green, with the average performance for each method in red. The performance across all 8 methods is demonstrated to be above 80%, with the best performing method (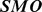) yielding classification performance of 99%.

Of these 50 methods, the 8 best performing (i.e. those that yielded an average Matthews' correlation coefficient of over 90%) were selected for application to Shakespeare. These included: *MultilayerPerceptron*; *SMO*; *IB1*; *IBk*; *LMT*; *FT*; *Logistic*; and *SimpleLogistic*. Utilising only these methods and considering only the 20 highest and 20 lowest CM_–_1 marker words for Shakespeare, a further 10-by-10 fold cross-validation was performed to model his authorship. These results, along with the mean performance for each method, are presented in Figure 14. Of the 168 plays in the text corpus dataset, the 28 authored by Shakespeare were classified with an average Matthew's correlation coefficient of over 90%, with the best performing method (*SMO*) yielding a coefficient of 99%.

### Performance Comparison of CM_–_1 and Welch's 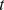-test

By following the same procedure described in the Materials and Methods section for the CM_–_1 score, a list of 20 high and 20 low scoring marker words may be generated for each author using the 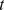-test. Table 4 presents these marker words for Shakespeare.

**Table 4:** 20 highest (left) and lowest (right) ranking words by Welch's 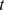-test score for Shakespeare, presented in descending and ascending order of score respectively.

Highest	Lowest
so[adverbManner]	all
speak	only
say	can
pluck	somewhat
amen	amongst
spoke	yes
hath	reach
oath	hopes
tonight	joy
answer	cast
wherefore	still
beseech	may
note	enjoy
go	must
ear	wrought
adieu	reward
did	private
brief	ease
purpose	clear
therefore	to[infinitive]

By considering the 5 best performing WEKA ensemble methods (*SMO*, *IB1*, *IBk*, *MultilayerPerceptron* and *Logistic*), the performance of models generated using CM1 score marker words can be compared directly to those generated using the equivalent 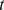-test marker words. These results are presented in Table 5, with performance evaluated in terms of Matthews' correlation coefficient (MCC), specificity (precision) and sensitivity (recall). It is evident that CM_–_1 yields a higher MCC in all examples.

**Table 5:** Performance results for the top 5 WEKA models, from the ensemble selected for Shakespeare authorship attribution based on performance against Fletcher, Jonson and Middleton (see Materials and Methods section).

	CM1 Score			Welch's t-test		
Method	MCC	Spec.	Sens.	MCC	Spec.	Sens.
SMO	0.987	1.000	0.980	0.960	1.000	0.940
IB1	0.955	0.987	1.000	0.863	1.000	0.800
IBk	0.955	0.987	1.000	0.863	1.000	0.800
MultilayerPerceptron	0.934	0.933	0.920	0.930	0.993	0.920
Logistic	0.923	0.960	0.980	0.916	0.967	0.960

Performance is evaluated in terms of Matthews' correlation coefficient, specificity (precision) and sensitivity (recall) for marker words selected by both CM1 and Welch's 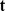-test scores.

As the ensemble of best performing WEKA models may differ when repeating the experimental procedure with the 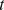-test as a method of marker selection, Table 6 provides a direct performance comparison between the overall 5 best performing models from the full set of 50 WEKA methods available. It is evident that the 3 best performing CM_–_1 models yield a higher MCC than any generated from 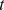-test marker words.

**Table 6:** Performance results for the top 5 WEKA models for both CM1 and Welch's 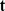-test scores, evaluated in terms of Matthews' correlation coefficient, specificity (precision) and sensitivity (recall).

CM_–_1 Score				Welch's t-test			
Method	MCC	Spec.	Sens.	Method	MCC	Spec.	Sens.
SMO	0.987	1.000	0.980	NaiveBayes	0.963	1.000	0.940
NaiveBayes	0.979	0.987	1.000	NaiveBayes	0.963	0.987	0.980
				Updateable			
NaiveBayes	0.979	0.987	1.000	Simple	0.960	1.000	0.940
Updateable				Logistic			
IB1	0.955	0.987	1.000	SMO	0.960	1.000	0.940
IBk	0.955	0.987	1.000	LMT	0.960	1.000	0.940

## Discussion

Given a large dataset, such as the text corpus of 168 plays from the Shakespearean era, a commonly accepted method of filtering the data to facilitate classification is Welch's 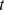-test. This score treats the two sets of observations as homogenous overall; an assumption that does not apply when attempting to identify play authorship, where one set contains the work of a single author, and the other contains the combined works of many. Instead of moderating by the standard deviation of the combined set, a new score (the CM_–_1 score) is introduced, with a moderator that considers the overall range of values of the larger set. The CM_–_1 score, in addition to facilitating the selection of marker words that yield authorship classification performance of over 90% (in terms of Matthews' correlation coefficient), has demonstrated a remarkable agreement with previously published observations.

The magnitude of CM_–_1 scores for Shakespeare's dominant negative markers is greater than that of his positive. Furthermore, the overall range of values for Shakespeare is comparatively small. This supports previous research suggesting that Shakespeare generally adheres to the norms of the work of his peer group [30]. Similarly, ‘
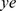
’ is shown to dominate as a positive marker for Fletcher, supporting Hoy's earlier observation that this word is characteristically overused in his plays [21].

Although the presented classification results demonstrate the CM_–_1 score as a powerful new method in the identification of individualising markers, the experimental method includes one simplification of the problem. The 20 highest and 20 lowest scoring marker words used for classification have been determined by considering the entire text corpus dataset, inclusive of each play as it is classified. To ensure that this method is able to generalise effectively to unencountered plays, a 10-by-10 fold cross validation was performed, with the frequency of each of the 55,055 individual words occurring as a high or low marker calculated across 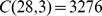 combinations of plays. This corresponds with the removal of every combination of 10% of plays by Shakespeare (3 plays), and for each, the removal of a random selection of 10% of plays by other authors (14 plays). Considering every possible selection of 14 plays by other authors would result in 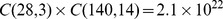 total combinations, which is infeasible to calculate.

**Figure 15:**
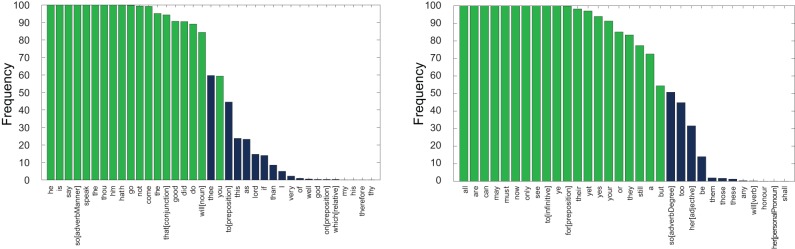
Frequency of occurrence of words appearing among the 20 highest scoring marker words for Shakespeare, resulting from a 10 fold cross validation. This process involved the removal of 10% of plays by Shakespeare (3), and 10% of plays by other authors (14). The 20 highest (left) and lowest (right) scoring marker words were calculated for every possible triplet of removed plays by Shakespeare (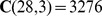 combinations), and for each, a random selection of 14 plays by other authors. The marker words determined across the full text corpus are highlighted in green. This demonstrates this selection of words as valid for classification, and that the CM_–_1 score is robust against the removal and addition of plays.

Figure 15 demonstrates the frequency of each of the 55,055 individual words occurring as one of the 20 highest (left) and lowest (right) scoring markers, for all words with nonzero occurrence. The marker words determined across the full text corpus are highlighted in green. This demonstrates the robustness of this selection of marker words against the removal and addition of plays (with the exception of ‘you’, which was 0.4% less likely to occur as a positive marker than ‘thee’).

The authorship results presented suggest that authors' individual styles are distinctive to a quantifiable degree. The rates at which they use some of the most common words in the language are consistently different from each other, and when used together serve to model characteristic styles in authorship. This finding supports arguments regarding the importance of the idiolects of individual language users [31].

### Weakly Attributed Plays

**Figure 16:**
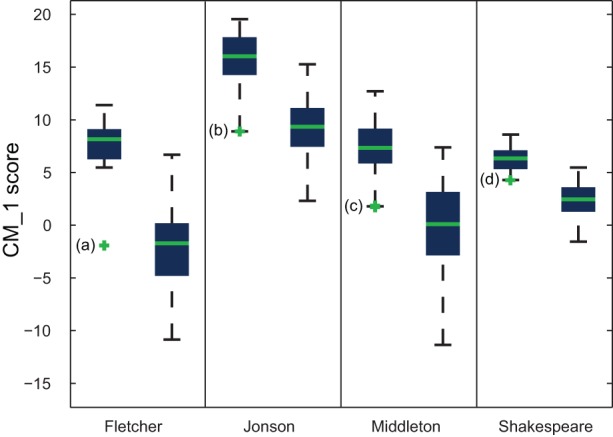
Difference between the cumulative CM_–_1 scores for the 20 highest and 20 lowest scoring marker words for Fletcher, Jonson, Middleton and Shakespeare. For each author, the left box represents the distribution of scores for their plays, and the right box the distribution of scores for plays by all other considered authors. The worst scoring play belonging to each author is indicated by a green cross. These are: a) *The Faithful Shepherdess* (Fletcher); b) *The Case is Altered* (Jonson); c) *A Game at Chess* (Middleton); and d) *Love's Labour's Lost* (Shakespeare).

Considering the 20 highest and 20 lowest CM_–_1 scoring marker words for Middleton (see Tables 2 and 3), Figure 8 demonstrates the ability of this score to identify plays of his authorship. The observed dip in performance corresponds with the play *A Game at Chess*, as indicated in Figure 16. (The version of *A Game at Chess* considered is the manuscript belonging to Trinity College, Cambridge, in Middleton's hand (MS. 0.2.66).)Although there is no doubt Middleton wrote this play, it is unusual stylistically among his works, being a satire on contemporary international politics, allegorised in the form of a chess game [32], [33]. Furthermore, eight of the marker words appearing in Tables 2 and 3 (‘
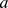
’, ‘
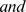
’, ‘
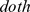
’, ‘
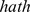
’, ‘
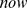
’, ‘
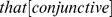
’, ‘
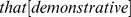
’ and ‘
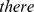
’) appear among the ten word-variables listed by Craig as the result of a discriminant analysis of Middleton's plays [34].

Although performance outliers may be explained by a work being of a different genre, chronology may also be a contributing factor. As highlighted by one reviewer, *A Game at Chess* is Middleton's last play; it was completed just three years before his death at age 44. Future applications of the CM_–_1 score may include investigating the feasibility of temporal models of an author's unique style, rather than treating it as homogenous over time.

Among Jonson's plays, the poorest attribution by a significant margin is that of *The Case is Altered*, as indicated in Figure 16. This play is generally regarded as an anomaly stylistically among Jonson's works. It is a romantic comedy, while Jonson's comedies are generally satirical. Jonson did not include it in his volume of collected works, and scholars have sometimes suggested that it is a collaboration [35]. Something similar holds for Fletcher's *The Faithful Shepherdess*, which is the only one of his plays to score negatively in Figure 4. This play is generally considered to be of a significantly different genre to the remainder of Fletcher's plays, and has been omitted in two previous studies attempting to identify his stylistic signature [21], [36].

### Maxwell's Demon

If language and the potential of words are considered as an abstract chaotic system with a high entropy, then the art and process of authorship within the structural and grammatical bounds of language results in a reduction of the original system's entropy. The presented results demonstrate that different authors, while adhering to the bounds of language, reduce the entropy of the system in characteristically different and measurable ways. Perhaps a good model to describe this individual behaviour of authorship is *Maxwell's Demon*, introduced by James Clerk Maxwell in a letter he wrote to Peter Guthrie Tait in 1867 [37].

The Second Law of Thermodynamics suggests that any process in a system will tend to increase the entropy of the universe. Two gases, one warmer than the other, brought into contact with each other, will always move towards equilibrium in temperature. In a letter of 1867 [37], James Clerk Maxwell formulated a thought experiment to illustrate how an exception to this law could be conceived. He pictured a ‘demon’ with superhuman powers, operating at a passage between two chambers. Its speed and facility allows it to follow the motion of molecules, and act quickly enough to let only faster molecules into one chamber, and only the slower moving molecules into the other. Without any apparent expenditure of effort, the two temperatures move further apart, in contradiction to the Second Law.

Whatever the cogency of the perceived contradiction, this celebrated and much debated scientific fable provides an analogy for the processes of language production as they emerge from a computational analysis of writing style. All authors draw on the common elements of a given language, sharing with their audience a set of vocabulary items and an established set of implicit but firm rules for combining these items. Yet each author makes an individual selection from common vocabulary and, while remaining with the rules of grammar, follows characteristic, finely differentiated patterns in phrase and sentence structure. Each author, in other words, starts from a vast, inchoate set of potential utterances and brings to it the order of an individual style. Without apparent effort, in a largely automatic process, entropy is reduced.

The described process must be faster than conscious thought, given the complexity of the task and the constraints of spontaneous production. Computational stylistics demonstrates this process in motion. Language individuation is multi-layered and subtle, but it leaves traces in simple frequencies, which can be monitored. As the user turns the common resources of a language into a personal discourse, some words in the language stream emerge at rates above the norm of similar language samples, and some are avoided entirely or otherwise substantially filtered. Maxwell's Demon is ‘finite’, yet unthinkably ‘sharpened’ in its ‘faculties’, recognising and dividing a swarm of individual, rapidly moving molecules with uncanny skill. Computational stylistics allow the observation of something similar is language; patterns of enhancement and suppression of the flows of very common words.

## Conclusion

Maxwell's Demon effortlessly reduces entropy by identifying and sorting atoms at superhuman speeds, thus apparently defying the second law of thermodynamics. This proves to be a good analogy for the process by which individual language users make a consistent and distinctive idiolect from the language available to them, a process which occurs at the level of the very common function words as well as in the more noticeable lexical words. Experiments with word-variables chosen with the aid of a new score, designed to identify marker variables by comparing means of two heterogeneous groups of specimens (where one group is much more mixed than the other) demonstrate that a small set of very common words provides markers which can help separate plays into authorial groups at a high level of reliability. The results show that words used at a lower rate than the aggregate of other authors are just as useful as words used at a higher rate.

The new score, CM_–_1, is adapted to the situation common in authorship problems where specimens need to be compared to a single authorial group on the one hand, and a mixed group of other authors on the other. The denominator for the difference in means is the range for the more mixed group rather than the standard deviation of the combined set, as with the 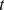-test. In the tests, markers identified by CM_–_1 out-perform those provided by Welch's 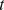-test. These results have implications for the understanding of individual differences in language, while the new score, the focus on the commonest variables, and the equal attention paid to under-utilised words all have implications for future authorship work. Future applications may also be found in other areas where there are very large numbers of possible marker variables available, such as the areas of transcriptomics, proteomics and other -omics, which are characterised by the use of high-throughput technologies.

## Supporting Information

File S1 **Complete text corpus dataset (i.e. the frequencies of 55,055 unique words for 168 Shakespearean-era plays).**
(ZIP)

File S2 **Reference figure combing** **Figures 3** **–** **10** **for side-by-side comparison.**
(ZIP)
